# Predominance of HIV-1 subtype A and D infections in Uganda.

**DOI:** 10.3201/eid0606.000609

**Published:** 2000

**Authors:** D J Hu, J Baggs, R G Downing, D Pieniazek, J Dorn, C Fridlund, B Biryahwaho, S D Sempala, M A Rayfield, T J Dondero, R Lal

**Affiliations:** Centers for Disease Control and Prevention, Atlanta, Georgia 30333, USA. dhu@cdc.gov

## Abstract

To better characterize the virus isolates associated with the HIV-1 epidemic in Uganda, 100 specimens from HIV-1-infected persons were randomly selected from each of two periods from late 1994 to late 1997. The 200 specimens were classified into HIV-1 subtypes by sequence- based phylogenetic analysis of the envelope (env) gp41 region; 98 (49%) were classified as env subtype A, 96 (48%) as D, 5 (2.5%) as C, and 1 was not classified as a known env subtype. Demographic characteristics of persons infected with the two principal HIV-1 subtypes, A and D, were very similar, and the proportion of either subtype did not differ significantly between early and later periods. Our systematic characterization of the HIV-1 epidemic in Uganda over an almost 3-year period documented that the distribution and degree of genetic diversity of the HIV subtypes A and D are very similar and did not change appreciably over that time.


### Predominance of HIV-1 Subtype A and D Infections in Uganda

**Dale J. Hu,* James Baggs,† Robert G. Downing,*† Danuta Pieniazek,* Jonathan Dorn,* Carol Fridlund,* Benon Biryahwaho,‡ Sylvester D.K. Sempala,‡ Mark A. Rayfield,* Timothy J. Dondero,* and Renu Lal***

*Centers for Disease Control and Prevention, Atlanta, Georgia, USA; †State of Washington, Department of Labor and Industries, Olympia, Washington, USA; ‡Uganda Virus Research Institute/ Centers for Disease Control and Prevention Research Collaboration, Uganda Virus Research Institute, Entebbe, Uganda

To better characterize the virus isolates associated with the HIV-1 epidemic in Uganda, 100 specimens from HIV-1-infected persons were randomly selected from each of two periods from late 1994 to late 1997. The 200 specimens were classified into HIV-1 subtypes by sequence-based phylogenetic analysis of the envelope (*env*) gp41 region; 98 (49%) were classified as *env* subtype A, 96 (48%) as D, 5 (2.5%) as C, and 1 was not classified as a known *env* subtype. Demographic characteristics of persons infected with the two principal HIV-1 subtypes, A and D, were very similar, and the proportion of either subtype did not differ significantly between early and later periods. Our systematic characterization of the HIV-1 epidemic in Uganda over an almost 3-year period documented that the distribution and degree of genetic diversity of the HIV subtypes A and D are very similar and did not change appreciably over that time.

HIV strain characterization and phylogenetic analysis have played a key role in elucidating epidemiologic and historical aspects of HIV transmission worldwide (1). In Thailand, for example, molecular analyses of virus isolates documented the independent introduction and spread of two different HIV-1 subtypes, B and E, as well as the increasing proportion of subtype E relative to B in injection drug users over time (2-8). Information from these studies clarified the dynamics of the Thai epidemic and influenced the decision to introduce a bivalent subtype B/E vaccine in the first HIV-1 vaccine efficacy trials recently initiated there (6). However, few other studies have described HIV-1 strains or the phylogenetic relationships of sequences from infections sampled in a systematic manner. This is especially true in central Africa, where the HIV epidemic has been present for a relatively long time (1-3,6,7,9-12).

Address for correspondence: Dale J. Hu, International Activities Branch, Division of HIV/AIDS Prevention, NCHSTP, Centers for Disease Control and Prevention, E-50, Atlanta, GA 30333, USA; fax: 404-639-4268; e-mail: dhu@cdc.gov.

Uganda, where the prevalence of HIV-1 is one of the highest in the world, has been a focus of research and intervention efforts, including vaccine development for the first vaccine trial in Africa. Previous studies documented HIV-1 subtypes A and D as important strains, with a limited distribution of other variants (13-19). The primary objective of our study was to characterize the recent virus isolates of the HIV-1 epidemic in Uganda by describing the distribution and genetic diversity of the principal HIV-1 subtypes.

### Methods

In an ongoing collaborative effort between the Uganda Virus Research Institute and the Centers for Disease Control and Prevention to study HIV-1 genetic diversity, isolates from a number of different clinical sites and counseling and testing centers in Uganda were collected and characterized (19). To obtain uniform and consistent sampling over time from the Kampala and Mpigi districts of central Uganda, where most specimens were collected, we selected two sites in Kampala (Mulago Hospital, Nsambya

Mobile Unit) and one site in Entebbe (Uganda Virus Research Institute Clinic). From more than 1,800 specimens collected from these sites from late 1994 through 1997, we chose all seropositive specimens from the earliest period (the last quarter of 1994 through the first quarter of 1995, n=393, and the last two quarters of 1997, n=135). After determining that the major demographic factors, such as mean age (p=0.76), age group (p=0.20), sex (p=0.12), and marital status (p=0.14), were not significantly different between persons seen in either period, we selected 100 specimens at random from each period for further analysis. Because local resources were limited, clinical (World Health Organization stage) and immunologic (CD4 and CD8 counts) data were available from only a subset of patients. Although limited by the number of specimens available and by laboratory capacity, our sample size could detect a minimum absolute change of more than 20% in either subtype A or D with a power of 80% and with 95% confidence.

Data collection, specimen processing, viral isolation, and polymerase chain reaction (PCR) amplification of the *env* gp41 fragment (460 bp) used methods previously described (19-21). After amplification, DNA from the nested PCR was cycle-sequenced (60 ng DNA per sequencing reaction) with the ABI PRISM Dye Terminator Cycle Sequencing Ready Reaction Kit (Perkin-Elmer, Foster City, CA), according to manufacturer's protocol, by using the PCR nested primers gp46F2 and gp47R2 (21).

### Results

Of all 200 specimens, 98 (49%) were classified as *env* subtype A, 96 (48%) as D, 5 (2.5%) as C, and 1 unclassified into a known *env* subtype category. The demographic characteristics of persons infected with the two principal HIV-1 subtypes, A and D, were very similar, and the proportion of either subtype did not differ significantly between early and later periods (Table 1). In addition, there were three subtype C isolates and one isolate of undetermined subtype in the early period and two subtype C isolates in the later period.

The Figure illustrates the phylogenetic trees for all HIV-1 *env* subtype A and D isolates. The mean pairwise intrasubtype nucleotide divergence for *env* gp41 was 7.8% (3.6%-22.3%) for subtype A specimens and 7.2% (2.8%-12.5%) for subtype D specimens. The translated amino acid regions of gp41 revealed only minor substitutions in subtypes A and D (data not shown). When specimens were further stratified by period and clinic, intrasubtype divergence of the two subtypes was similar, with the percentage of divergence slightly higher in the later period (Table 2). There was no clear segregation or clustering by period or locale (Figure).

Table 1. Demographic characteristics of persons infected with HIV-1 subtype A or D

| Characteristics | Subtype A n=98 | Subtype D n=96 | |
|---|---|---|---|
| Period | | | |
| Early (1994/95) | n=51 | n=45 | p=0.47 |
| Late (1997) | n=47 | n=51 | |
| Age (yrs) | | | |
| Median | 30.0 | 28.0 | |
| Mean | 31.2 | 30.8 | p=0.39 |
| Sex | | | |
| Female | 66% | 73% | p=0.26 |
| Male | 34% | 27% | |
| Marital status | | | |
| Married | 37% | 40% | p=0.66 |
| Single, divorced, widowed | 63% | 60% | |
| Clinic (district) | | | |
| UVRI[a] (Mpigi) | 37% | 33% | p=0.81 |
| Mulago (Kampala) | 23% | 24% | |
| Nsambya (Kampala) | 40% | 43% | |

[a]UVRI = Uganda Virus Research Institute.

In the subset of persons with clinical or immunologic data, the clinical characteristics of persons infected with *env* subtype A or D were very similar; any differences were not significant (Table 3).

### Discussion

Our detailed and systematic characterization of the HIV-1 epidemic in Uganda over an almost 3-year period has shown that the distribution and degree of genetic diversity of the two predominant *env* subtypes, A and D, are very similar and have not changed appreciably over that time. However, there are two caveats when interpreting our results. First, our sample was collected cross-sectionally without known dates of infection. Second, our available sample size was not large enough to document small changes in the proportion of either A or D subtypes. Nevertheless, the proportion of those two subtypes observed in specimens from the two periods were virtually identical.

Table 2. Mean (range) percentage of pairwise nucleotide divergence stratified by time period, subtype, and clinic (district)

| Subtype by clinic (district) | % Mean (range) 1994-95 | 1997 | Overall |
|---|---|---|---|
| Subtype A[a] | | | |
| UVRI[b] (Mpigi) | 5.0 (3.6- 9.5) | 7.7 (3.9-12.6) | 6.4 (3.6-12.6) |
| Mulago (Kampala) | 7.3 (3.9-10.4) | 8.3 (4.2-14.0) | 7.8 (3.9-14.0) |
| Nsambya (Kampala) | 7.3 (3.9-11.7) | 10.8(3.9-22.3) | 9.1 (3.9-22.3) |
| Subtype D | | | |
| UVRI (Mpigi) | 5.9 (3.1- 8.8) | 8.0 (2.8-12.4) | 6.9 (2.8-12.4) |
| Mulago (Kampala) | 7.9 (3.6-12.4) | 6.7 (3.1- 9.8%) | 7.2 (3.1-12.4) |
| Nsambya (Kampala) | 6.4 (3.3- 9.5) | 8.5 (3.9-12.5) | 7.4 (3.3-12.5) |

[a]Since two isolates from 1994-1995 were almost identical and may represent persons who were epidemiologically linked, these were treated as one sequence for overall calculations.
[b]UVRI = Uganda Virus Research Institute.

Table 3. Clinical characteristics among a subset of persons infected with HIV-1 subtype A or D[a]

| Characteristic WHO[a] clinical | Subtype A n=37 | Subtype D n=46 | p value |
|---|---|---|---|
| Stage | | | |
| 1 or 2 | 8% | 9% | p=0.97 |
| 3 | 57% | 54% | |
| 4 (AIDS) | 35% | 37% | |
| CD4 | n=29 | n=26 | |
| Mean | 253 | 187 | p=0.24 |
| Range | 19-1,061 | 2-713 | |
| CD8 | n=29 | n=26 | |
| Mean | 812 | 734 | p=0.45 |
| Range | 288-2,000 | 139-1,458 | |

[a]WHO = World Health Organization.

Our results are consistent with those of previous studies indicating that both subtypes A and D have been present as major strains in the Ugandan epidemic since the mid-1980s (13-17). The similar mean and range of intrasubtype variation for subtypes A and D in the *env* gp41 region in this report, as well as in the *env* C2-V3 we reported earlier (19), also suggest that the two predominant HIV-1 subtypes have both been present in Uganda for a relatively long period.

The lack of significant clustering by period or clinic in the phylogenetic analyses suggests that infections from both subtypes are transmitted in a broad, heterogeneous manner in central Uganda. Similarly, our examination of a subset of patients with clinical or immunologic data, in combination with similar overall demographic characteristics, suggests that persons with subtype A or D had similar levels of immune suppression. Since this information was independently collected at three sites before genetic characterization of infecting HIV-1 strains, a systematic selection bias with respect to subtype appears unlikely.

Although we did not document any major recent changes in the proportion of subtypes A or D, our ongoing surveys may show future changes in subtype distribution. In contrast to the large proportion of subtypes A and D, the proportion of subtype C appeared to be small in both periods studied and in our earlier study (19). The minor, nonexpanding, role of subtype C in central Uganda contrasts with the higher proportions of C in southeastern Africa. Recently, we reported that subtype C isolates from Uganda formed a distinct subcluster, unlike subtype C isolates from other locales (24).

The presence or absence of significant changes in subtype distribution in different populations is ecologic and by itself does not constitute definitive evidence for or against potential subtype-specific differences in transmissibility, pathogenesis, or clinical symptoms (25). In Uganda, where subtypes A and D are found in a large proportion of the population, current and future prospective studies will help to address these crucial research questions.

Although few countries, especially those with limited resources, have the necessary infrastructure to systematically monitor the HIV-1 epidemic, periodic molecular analyses such as this one in Uganda are needed, particularly if trials of subtype-specific vaccines are being considered. Since the HIV-1 pandemic is becoming increasingly complex, with greater heterogeneity and the presence of recombinant viruses (26), a major challenge will be to develop cost-effective technologies to characterize viruses without sequencing (1). While more rapid typing methods are available to determine the subtype distribution (19), we needed the labor-intensive sequencing and phylogenetic analysis in this study to describe the genetic diversity in detail.

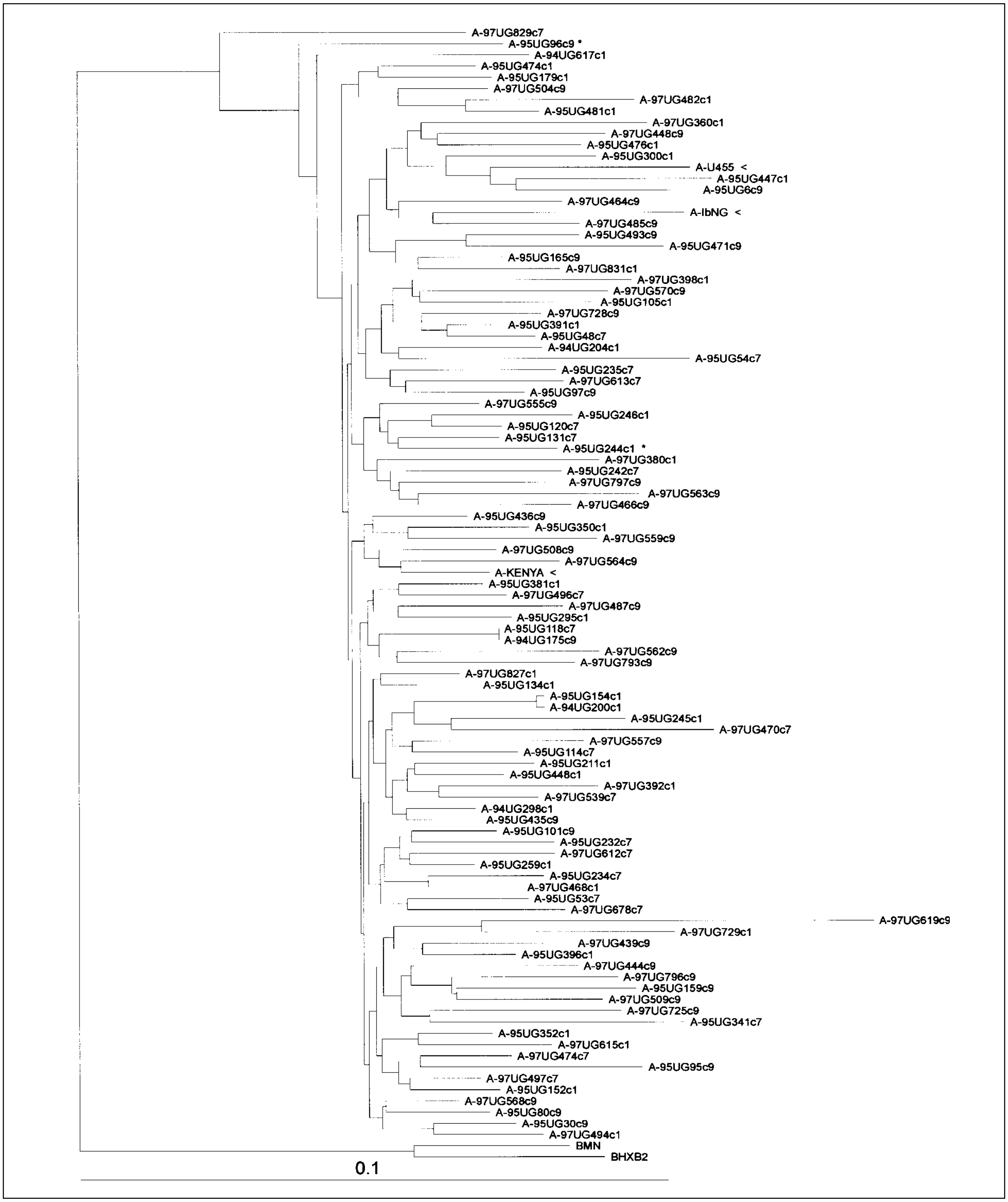

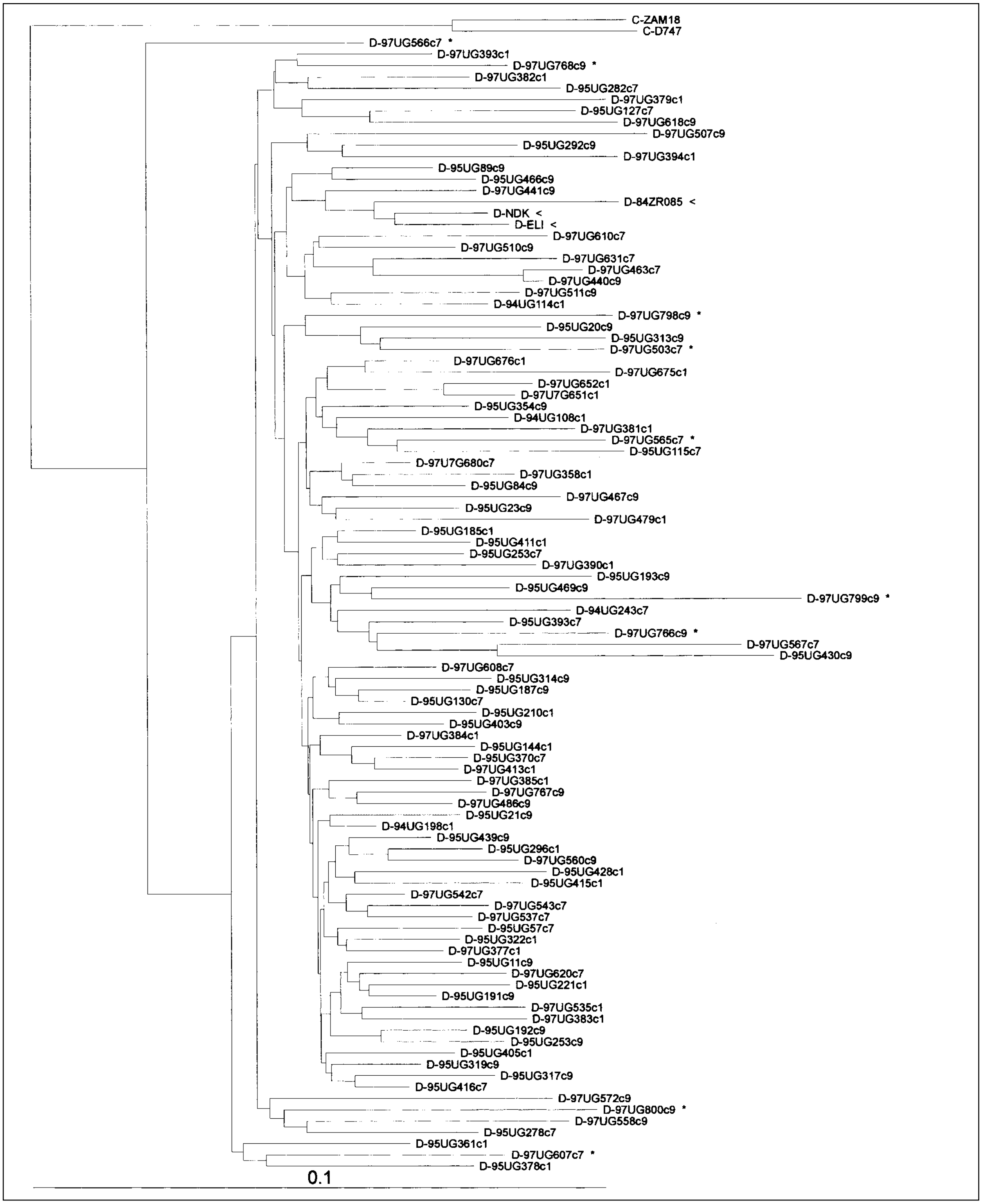

Figure. Phylogenetic classification of *env* gp41 HIV-1 sequences from Ugandan (UG) patients (GenBank accession numbers for subtypes A and D are pending). Numbers before the abbreviation UG indicate the year of specimen collection; c1, c7, and c9 denote the UVRI, Mulago, and Nsambya clinics, respectively. The trees were constructed on the basis of 354-bp DNA sequences by the neighbor-joining method with nucleotide distance datum sets calculated by Kimura's two-parameter approach and rerooted by using SIV-cpz as the outgroup. Arrows indicate reference subtype A and D sequences; asterisks indicate sequences, which decrease the bootstrap value from 90% to 73% in subtype A and from 85% to 55% in subtype D sequences. The scale bar indicates the evolutionary distance of 0.10 nucleotides per position in the sequence. Vertical distances are for clarity only. An automated DNA sequencer (Applied Biosystems Model 373, Foster City, CA) was used to generate sequence data for alignment with the CLUSTAL version V multiple sequence alignment program and subsequent phylogenetic analysis. Phylogenetic relationship of sequences was analyzed by the neighbor-joining method (PHYLIP package version 3.5c with and without bootstrapping), and the maximum-likelihood method (fastDNA program, version 1.0.8, which uses randomized data input and global rearrangement). The stability of tree topology was tested by pruning, which consisted of removing one species from the alignment and rerunning the phylogenetic analysis. Accurate subtype determination using *env* gp41 has been shown to be similar to that based on *env* C2V3 sequences (22). The gp41 DNA sequences Environment package, and immunodominant regions were analyzed (23). The reference sequences for subtypes A-J, groups O and N, and SIVcpz were retrieved from the 1997 HIV-1 Molecular Immunology Database (Los Alamos National Laboratory, Los Alamos, NM).

Furthermore, given the time and resources necessary to develop vaccine candidates, confirmation from this and previous studies that subtypes A and D continue to be the predominant subtypes with consistently minor contributions from other strains may have important implications for vaccine research.

### Acknowledgments

We thank the clients and staff of the Uganda Virus Research Institute in Entebbe, especially Gibson Anyaegani, Steven Balinandi, and Dennis Olara, for technical services; the Mulago Hospital and Nsambya Mobile Unit in Kampala; and the reviewers of this article for their thoughtful and helpful comments.

Dr. Hu is a medical epidemiologist at the Centers for Disease Control and Prevention in Atlanta, Georgia. His research interests include the impact of viral and host genetic variation on the molecular epidemiology and pathogenesis of HIV-1.

### References

1. Hu DJ, Dondero TJ, Rayfield MA, George JR, Schochetman G, Jaffe HW, et al. The emerging genetic diversity of HIV: The importance of global surveillance for diagnostics, research, and prevention. JAMA 1996;275:210-16.
2. Ou CY, Takebe Y, Luo CC, Kalish ML, Auwanit W, Bandea C, et al. Wide distribution of two subtypes of HIV-1 in Thailand. AIDS Res Hum Retroviruses 1992;8:1471-2.
3. Weniger BG, Limpakarnjanarat K, Ungchusak K, Thanprasertsuk S, Choopanya K, Vanichseni S, et al. The epidemiology of HIV infection and AIDS in Thailand. AIDS 1991;5:S71-S85.
4. McCutchan FE, Hegerich PA, Brennan TP, Phanuphak P, Singharaj P, Jugsudee A, et al. Genetic variants of HIV-1 in Thailand. AIDS Res Hum Retroviruses 1992;8:1887-95.
5. Wright NH, Vanichseni S, Akarasewi P, Wasi C, Choopanya K. Was the 1988 HIV epidemic among Bangkok's injecting drugs users a common source outbreak? AIDS 1994;8:529-32.
6. Kalish ML, Baldwin A, Raktham S, Wasi C, Luo CC, Schochetman G, et al. The evolving molecular epidemiology of HIV-1 envelope subtypes in injecting drug users in Bangkok, Thailand: implications for HIV vaccine trials. AIDS 1995;9:851-7.
7. Wasi C, Herring B, Raktham S, Vanichseni S, Mastro TD, Young NL, et al. Determination of HIV-1 subtypes in injecting drug users in Bangkok, Thailand, using peptide-binding enzyme immunoassay and heteroduple mobility assay: evidence of increasing infection with HIV-1 subtype E. AIDS 1995;9:843-9.
8. Subbarao S, Limpakarnjanarat K, Mastro TD, Bhumisawasdi J, Warachit P, Jayavasu C, et al. HIV-1 in Thailand, 1994-1995: persistence of two subtypes with low genetic diversity. AIDS Res Hum Retroviruses 1998;14:319-27.
9. Janssens W, Buve A, Nkengasong JN. The puzzle of HIV-1 subtypes in Africa. AIDS 1997;11:705-12.
10. Barin F, Courouce AM, Pillonel J, Buzelay L, Retrovirus Study Group of the French Society of Blood Transfusion. Increasing diversity of HIV-1M serotypes in French blood donors over a 10-year period (1985-1995). AIDS 1997;11:1503-8.
11. Muller-Trutwin MC, Chaix ML, Letourneur F, Begaud E, Beaumont D, Deslandres A, et al. Increase of HIV-1 subtype A in Central African Republic. J Acquir Immune Defic Syndr 1999;21:164-1.
12. Mastro TD, Kunanusont C, Dondero TJ, Wasi C. Why do HIV-1 subtypes segregate among persons with different risk behaviors in South Africa and Thailand? AIDS 1997;11:113-6.
13. Oram JD, Downing RG, Roff M, Serwankambo N, Clegg JCS, Featherstone AS, et al. Sequence analysis of the V3 loop regions of the env genes of Ugandan human immunodeficiency proviruses. AIDS Res Hum Retroviruses 1991;1:605-14.
14. Albert J, Franzen L, Jansson M, Scarlatti G, Kataaha PK, Katabira E, et al. Ugandan HIV-1 V3 loop sequences closely related to the U.S./European consensus. Virology 1992;190:674-81.
15. WHO Network for HIV Isolation and Characterization. HIV type 1 variation in World Health Organization-sponsored vaccine evaluation sites: genetic screening, sequence analysis, and preliminary biological characterization of selected viral strains. AIDS Res Hum Retroviruses 1994;10:1327-43.
16. Bruce C, Clegg C, Featherstone A, Smith J, Biryahawaho B, Downing R, et al. Presence of multiple genetic subtypes of human immunodeficiency virus type 1 proviruses in Uganda. AIDS Res Hum Retroviruses 1994;10:1543-50.
17. Smith JD, Bruce CB, Featherstone AS, Downing RG, Biryahawaho B, Clegg JCS, et al. Reactions of Ugandan antisera with peptides encoded by V3 loop epitopes of human immunodeficiency virus type 1. AIDS Res Hum Retroviruses 1994;10:577-83.
18. Brennan CA, Lund JK, Golden A, Yamaguchi J, Vallari AS, Phillips JF, et al. Serologic and phylogenetic characterization of HIV-1 subtypes in Uganda. AIDS 1997;11:1823-32.
19. Rayfield MA, Downing RG, Baggs J, Hu DJ, Pieniazek D, Luo CC, et al. A molecular epidemiologic survey of HIV in Uganda. AIDS 1998;12:521-7.
20. Luo CC, Downing RG, dela Torre N, Baggs J, Hu DJ, Respess RA, et al. The development and evaluation of a probe hybridization method for subtyping HIV type 1 infection in Uganda. AIDS Res Hum Retroviruses 1998;14:691-4.
21. Yang C, Pieniazek D, Owen SM, Fridlund C, Nkengasong J, Mastro TD, et al. Detection of phylogenetically diverse human immunodeficiency virus type 1 groups M and O from plasma by using highly sensitive and specific generic primers. J Clin Microbiol 1999;37:2581-6.
22. Pieniazek D, Yang C, Lal RB. Phylogenetic analysis of gp41 envelope of HIV-1 groups M, N, and O provides an alternate region for subtype determination. In: Korber B, Foley B, McCutchan F, Mellors JW, Hahn BH, Sodroski J, et al, editors. Human retroviruses and AIDS 1998. Los Alamos: Los Alamos National Laboratory;1998:III-112-17.

23. Dorn J, Masciotra S, Yang C, Downing R, Biryahwaho B, Mastro TD, et al. Analysis of genetic variability within the immunodominant epitopes of envelope gp41 from HIV-1 Group M and its impact on HIV-1 antibody detection. J Clin Microbiol 2000;38:773-80.
24. Downing R, Pieniazek D, Hu DJ, Biryahawaho B, Fridlund C, Rayfield MA, et al. Genetic characterization and phylogenetic analysis of HIV-1 subtype C from Uganda. AIDS Res Hum Retroviruses 2000;16:815-19.
25. Hu DJ, Buvé A, Baggs J, van der Groen G, Dondero TJ. What role does HIV-1 subtype play in transmission and pathogenesis? An epidemiological perspective. AIDS 1999;3:873-81.
26. Robertson DL, Sharp PM, McCutchan FE, Hahn BH. Recombination in HIV-1. Nature 1995;374:124-6.
